# Pre-ictal temperature increases detected by ear canal thermometry in the epilepsy monitoring unit. An exploratory study

**DOI:** 10.1016/j.cnp.2025.07.006

**Published:** 2025-08-05

**Authors:** Masud Seyal, Todd Chatlos, George Savvides, Shari Barela

**Affiliations:** Department of Neurology, University of California, Davis, USA

**Keywords:** Preictal. epitympanic, Temperature rise, Focal seizures, Epilepsy monitoring unit

## Abstract

**Objective:**

There is evidence for a pre-ictal state, distinct from the interictal state, that can be detected minutes before the onset of a clinical seizure. Analysis of scalp EEG signals do not reliably distinguish the pre-ictal EEG from the interictal EEG. Changes in cerebral blood flow and oxygenation occur several minutes before EEG evidence of a seizure. Local increases in cerebral activity are associated with increases in brain temperature. Brain temperature changes are reflected in the tympanic membrane temperature. This exploratory study investigated whether a brain temperature change could be detected noninvasively in the immediate pre-ictal period.

**Methods:**

Patients with focal seizures undergoing inpatient video-EEG telemetry had epitympanic temperatures recorded. Pre-ictal temperature changes deviating from the interictal temperature were studied.

**Results:**

Data was available for 25 seizures in 12 patients. For a given seizure, the mean temperature at seizure onset exceeded the interictal temperature by 0.31 °C. Peri-ictal peak temperature exceeded the interictal temperature by a mean of 0.37 °C. Duration of temperature rise was 1081 s.

**Conclusions:**

Pre-ictal temperature increases were detected non-invasively in focal onset seizures.

**Significance:**

This finding provides the basis for development of temperature-based technology for seizure warning in ambulatory patients with refractory epilepsy.

## Highlights

• Changes in blood flow and cerebral oxygenation occur prior to seizure onset.
• A pre-ictal temperature increase is detectable non-invasively in patients undergoing video-EEG telemetry.
• Our findings provide a basis for the development of temperature-based technology for seizure warning.

## 1 Introduction

For patients with uncontrolled epilepsy, the unpredictable nature of seizures is particularly disabling. Seizures carry a risk of serious injury and create a sense of helplessness that impacts daily life. If these patients had a reliable way to predict an impending seizure, it would give the patient a window for intervention that could improve their quality of life.

While progress has been made (Cook et al., 2013, Truong et al., 2018), current efforts using mathematical analysis of scalp EEG signals have not reliably distinguished the pre-ictal EEG from the interictal EEG (Mormann et al., 2007). There is non-EEG evidence indicating the presence of a pre-ictal state, distinct from the interictal state, that may be detected several minutes or hours before the onset of a clinical seizure. Changes in cerebral blood flow and oxygenation occurred several minutes before clinical and EEG evidence of a seizure (Adelson et al., 1999, Baumgartner et al., 1998, Federico et al., 2005, Seyal, 2013, Weinand et al., 1997). Functional MRI (fMRI) analysis in the immediate pre-ictal state showed a regional increase in the blood oxygen level-dependent (BOLD) signal (Federico et al., 2005). SPECT scans obtained in the immediate pre-ictal period (11 and 12 min before seizure onset) showed an increase in regional blood flow without detectable EEG change during that time (Baumgartner et al., 1998). In direct recordings over an epileptic focus, using subdural thermal diffusion flowmetry cerebral blood flow (CBF) probes for long term CBF monitoring, increased CBF began 20 min prior to clinical seizure onset in patients with temporal lobe epilepsy (Weinand et al., 1997). Using near-infrared spectroscopy, changes in regional cerebral oxygenation were detected 5 min prior to EEG seizure onset (Seyal, 2013). Transcranial magnetic pulse stimulation techniques showed increased cortical excitability in a 24-hour window prior to seizure onset (Badawy et al., 2009). None of these methods are likely to result in a clinically relevant tool for seizure prediction that could be of direct benefit to most patients with uncontrolled seizures. A practical tool would require a reliable signal of an imminent seizure that could be detected by a simple, affordable, and non-invasive means.

Local increases in cerebral activity are associated with increases in brain temperature (Kiyatkin et al., 2002). In rodents, local brain temperature increases of 1–1.8 °C occur in response to various novel, stressful, and emotionally arousing environmental stimuli (Kiyatkin et al., 2002). The local metabolic consequences of widely correlated neural activity appear to be the primary source of changes in brain temperature (Kiyatkin et al., 2002). In rodents and primates, experimentally induced focal seizures result in an approximately 0.3 °C increase in temperature (Torres et al., 2024, Torres et al., 2023, Yang et al., 2002).

Brain temperature changes are reflected in corresponding changes in tympanic membrane temperature as the middle ear is heated passively by the brain (Cherbuin and Brinkman, 2007). Tympanic membrane temperature is closely correlated with the temperature of the hypothalamus (Dickey et al., 1970). Lateralized changes in tympanic membrane temperature ranging between 0.9 and 1.656°C occurred in chimpanzees during various cognitive tasks (Hopkins and Fowler, 1998).

It remains to be determined whether any pre-ictal changes in brain temperature can be detected noninvasively in humans by measuring temperature in proximity to the tympanic membrane. The present exploratory study was specifically designed to evaluate whether, for a given seizure, there is a discernable pre-ictal temperature change that deviates from the immediately preceding interictal temperature. Longer duration circadian and ultradian brain temperature variations, not directly related to seizures, may occur and such long-term temperature changes were not the focus of the present study.

## 2 Materials and methods

The study was conducted in patients aged 18–65 years with suspected focal epilepsy, undergoing inpatient video-EEG telemetry (VET) at the University of California, Davis Medical Center (UCDMC) as part of their work up for possible epilepsy surgery or spell capture.

Patients with intracranial electrodes, history of progressive neurological disease, clinically significant concurrent medical or psychiatric illness, pregnant or lactating women, history of current heavy alcohol or illicit drug use, a history of ear canal or tympanic membrane pathology or those who were unable to provide informed consent were excluded. Patients unable to tolerate the temperature probe were excluded from the study. The study was approved by the UCDMC Institutional Review Board.

The external auditory canal was examined with an otoscope to ensure that there was no cerumen obstructing view of the tympanic membrane or other local pathology before placing of the temperature sensor. For the purposes of this study, we defined the interictal baseline temperature as the average of at least 2 min of temperature data obtained just prior to the onset of a visible rise in pre-ictal epitympanic temperature. In defining the baseline, it was ensured that there was artifact free recording (no abrupt rise or drop in temperature) and that there was no change in the state of the patient (such as transitioning between sleep to wakefulness on EEG). We excluded temperature data obtained while the patient was eating, talking, or when the patient’s ear with the temperature probe was resting on a pillow.

The duration of the pre-ictal rise in temperature was measured. We recorded the peak *peri*-ictal epitympanic temperature, measured within the external auditory meatus, immediately prior to or in proximity to electrographic seizure onset. The duration of the pre-ictal temperature was measured from when the temperature rose 2 standard deviations (s.d.) above the interictal baseline mean to when the electrographic seizure began. Temperature data that were not time-locked to seizure onset were not studied.

Standard protocols in effect at the UC Davis Medical Center epilepsy monitoring unit (EMU) for this study were followed. The current standard of care in the EMU was not altered for the purposes of this study. Standard scalp EEG recording sites (International 10–20 system) for video-EEG telemetry (VET) were used. Continuous synchronized video, EKG, nasal airflow (using a pressure transducer) and abdominal excursions (using band plethysmography) were recorded. Synchronous oxygen saturation values were recorded using digital pulse oximetry. Antiseizure medications were tapered down on an individualized basis, at the discretion of the attending epileptologist, to increase the likelihood of seizures occurring during the hospitalization. Patients were hospitalized until adequate seizure localizing information was obtained.

Epitympanic temperature was recorded using a thermister-based thermometer (Novamed USA) placed in the ear canal. For patient convenience, temperature was recorded from only one ear ipsilateral to the patient’s seizure localization as determined by the patient’s first seizure in the EMU. The temperature sensor was connected to a standard hospital patient vital sign monitor (Philips MX 450) displaying temperature changes with a resolution of 0.01°C and updated every second. The temperature data were recorded and stored on a computer for subsequent analysis. For patient convenience, when possible, we sought to remove the temperature probe after one seizure with concurrent temperature data had been captured, however, for logistical reasons, this was not possible in several patients.

Starting at EEG seizure onset, the immediately preceding temperature data obtained synchronously with the VET was analyzed for a change in epitympanic temperature. These data were examined in relation to the timing and localization of EEG seizure onset. Specifically, we determined whether there was a change in epitympanic temperature, relative to the patient’s interictal baseline, leading to the EEG onset of a seizure and evaluated the timing and magnitude of any such change. The duration of pre-ictal temperature change was defined as the period starting at the time at which the change in temperature exceeded 2 s.d. above the patient’s interictal mean temperature and ending at the electrographic onset of the seizure. Peri-ictal temperature deviations of less than 0.1 °C from baseline were considered to be unchanged.

## 3 Results

Temperature data was available for 25 seizures in 12 patients (5 female) (Table 1). The mean patient age was 39 years (range 31–62). When data from consented patients was partially lost due to technical and patient-related issues in the EMU, further analysis was not performed. Twelve seizures had a left temporal onset, six seizures had a right temporal onset, the remaining seizures had extratemporal onsets or onsets could not be localized. For 19 seizures the patient was awake at seizure onset, and for 6 seizures the patient was asleep prior to seizure onset. Four seizures proceeded to secondary generalization.

**Table 1:** Patient clinical data.

Pt #	Sex	Age years	Probe side	Baseline temp (°C)	Duration temp rise (Seconds) (onset to temperature peak)	Duration temperature rise (Seconds) (Onset to seizure onset)	Peri-ictal peak temperature (°C)	Temp increase (from baseline mean to *peri*-ictal temperature peak) (°C)	Temperature increase (from baseline mean to temperature at seizure onset) (°C)	Sz Lat	Sz Loc	Sz Dur	State	MRI	PET
1	M	32	L	35.54	584	435	35.64	0.1	0.36	L	T	85	S	N	
2	M	31	L	34.35	10,157	10,157	35.32	0.97	0.97	L	T	155	A	N	
3	M	44	L	33.48	1306	1805	33.90	0.42	0.42	L(G)	T	118		N	
4	M	42	L	34.75	240	743	34.90	0.15	0.15	L	T	60	A	ROFE	BF
			L	35.1	603	607	35.35	0.26	0.255	L	T	51	A		
			L	35.2	266	1578	35.57	0.37	0.49	L	T	44	S		
5	F	36	R	34.9	494	1339	35.07	0.17	0.07	B	BP	56	A	N	BT
			R	34.5	356	230	34.68	0.18	0.06	B	BP	79	A		
			R	34.47	148	−49	34.65	0.18	0.03	B	BP	59	A		
			R	34.53	258	115	34.76	0.23	0.01	B	BP	59	A		
6	F	35	L	30.25	569	561	30.48	0.24	0.23	L	T	109	A	N	BT
			L	33.19	545	598	33.30	0.11	0.14	L	T	70	A		
			L	32.84	627	918	33.16	0.32	0.25	L	T	104	A		
			L	30.25	629	621	30.48	0.23	0.22	L	T	109	A		
7	F	40	R	35.3	186	206	35.48	0.18	0.18	R	PQ	57	A	N	
			R	35.09	685	421	35.45	0.36	0.37	R	PQ	93	A		
8	F	62	R	32.51	113	113	32.97	0.46	−0.16	R	T	67	A	RMTS	RT
			R	33.51	454	454	34.26	0.75	0.73	R	T	70	A		
			R	33.03	2142	2142	33.37	0.34	0.75	R	T	70	A		
			R	33.38	230	230	34.09	0.71	0.69	R	T	85	A		
9	F	37	R	32.75	1346	1346	33.38	0.63	0.63	R(G)	T	186	S	N	RT
10	M	37	L	34.54	258	258	35.17	0.63	0.15	L	T	161	S	CVL	BF
11	M	36	R	35.52	340	340	36.24	0.72	0.11	R(G)	T	110	S	N	LT
12	M	37	L	35.28	766	766	35.68	0.32	0.32	L (G)	T	192	A	N	N

M = male, F = female; Sz lat = seizure lateralization; Sz loc = seizure localization; Sz dur = seizure duration (seconds); L = left; R = right; B = bilateral; G indicates that the seizure generalized; T = temporal; BP = bilateral parietal; PQ = posterior quadrant; A = patient awake at seizure onset; S = patient asleep at seizure onset; N = normal MRI; ROFE = right orbitofrontal encephalomalacia; RMTS = right mesial temporal sclerosis; CVL = cerebellar volume loss. For PET scans, BF = bifrontal hypometabolism; BT = bitemporal hypometabolism; RT = right temporal hypometabolism; LT = left temporal hypometabolism.

Data from one seizure of right frontal onset with no pre-ictal temperature increase and data from a bilateral parietal onset seizure with no temperature increase are excluded from the table.

**Fig. 1:**
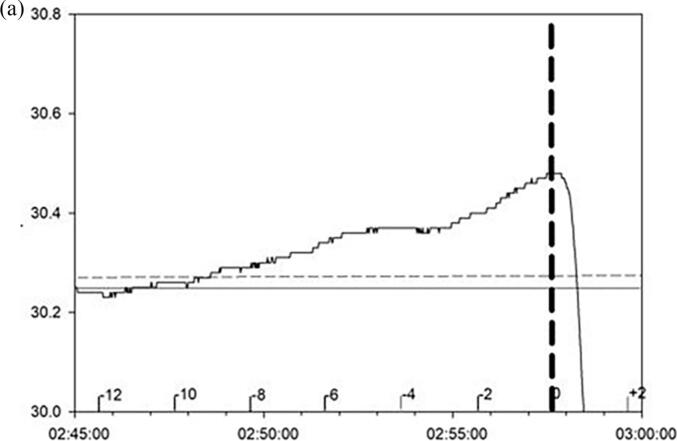

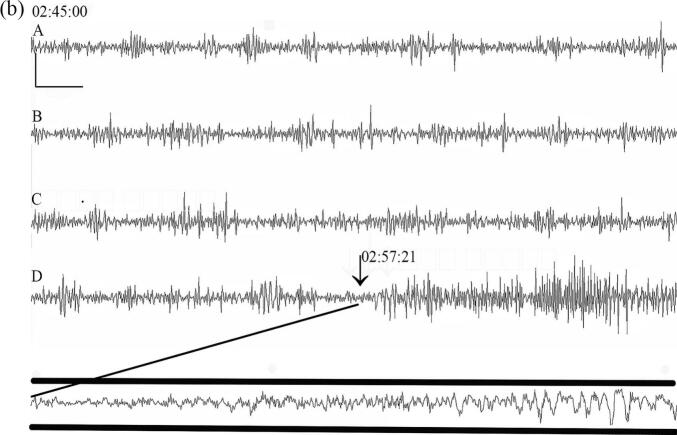
1a and 1b. Temperature graph and concurrent EEG with a focal seizure of temporal onset. 1a. Y-axis indicates temperature in °C. The solid horizontal line shows the mean interictal baseline temperature. The dashed horizontal line is at 2 s.d. above the mean baseline temperature. Seizure onset at 02:57:21 is indicated by the thick vertical dashed line. Time of day and minutes prior to seizure onset are indicated on the X-axis. The abrupt drop in temperature at or immediately following clinical seizure onset is related to the probe being dislodged from the ear canal. The temperature drops towards ambient room temperature. 1b. EEG obtained concurrently with temperature tracing in Fig. 1a. From top to bottom, four contiguous (A-D) EEG segments (tracing C3-T3) compressed to 2 mm per second starting at 02:45:00 that coincides with the preictal onset of temperature rise. Horizontal calibration bar 15 s. (EEG display gain at sensitivity of 5 µV per millimeter). The arrow points to seizure onset. The bottom EEG trace (T3-P3) that is bounded by the thick horizontal lines is expanded to better show seizure onset.

**Fig. 2:**
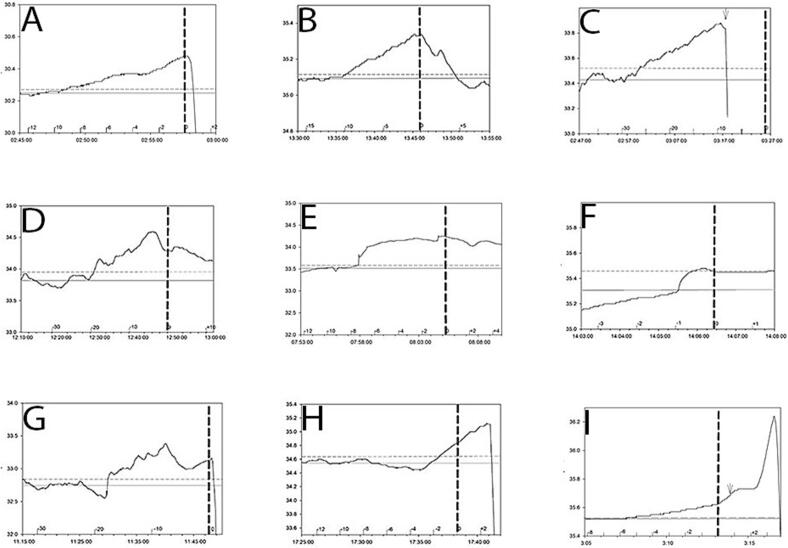
Pre-ictal epitympanic temperature. Nine representative seizures in nine patients. Seizure onsets are indicated by thick vertical dashed lines. X-axis: Time of day and time preceding seizure onset (minutes). Y-axis: Temperature in °C. The solid horizontal lines depict the mean temperature of the interictal baseline, the dashed horizontal lines are at + 2 s.d. above the mean interictal baseline. Abrupt drops in temperature in traces A, C, G and H and I that occurred at or immediately following seizure onset coincide with the temperature probe being dislodged from the ear canal. Note that in trace I the temperature continues to rise after seizure onset until the probe is dislodged.

The temperature at electroclinical seizure onset was higher than the patient’s mean interictal baseline temperature by an average of 0.31 ± 0.28 °C s.d. (range −0.16–0.97). The *peri*-ictal peak temperature was higher than the patient’s pre-ictal interictal epitympanic temperature by a mean of 0.37 ± 0.24 °C (range 0.1––0.97). In this group of patients, the mean interictal baseline epitympanic temperature across seizures was 33.97 ± 1.46 °C (range 30.25 to 35.54) (Fig. 1, Fig. 2, Table 1).

The mean duration of recorded pre-ictal temperature rise above baseline (starting when the temperature exceeded 2 s.d. above interictal baseline and ending at seizure onset) was 1081 s (range −49 to 10,157 s). The mean rate of rise of temperature (baseline to peak) was 0.05°C / minute.

**Fig. 3:**
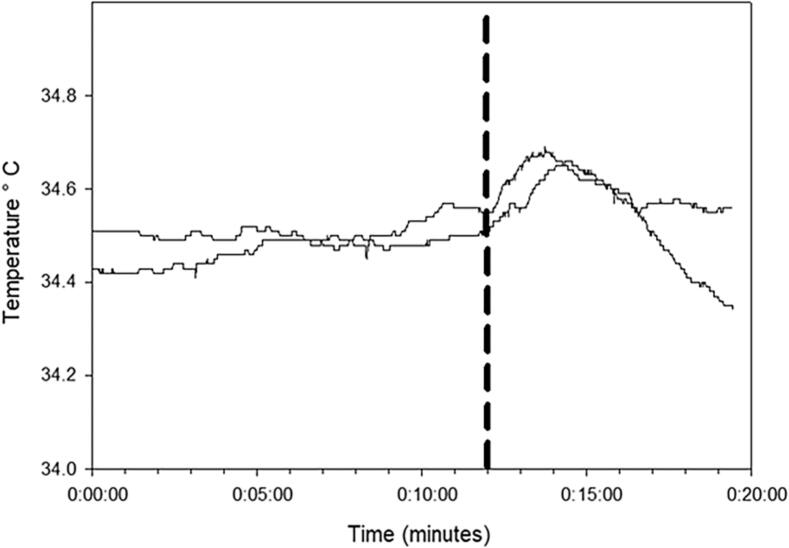
Reproducibility of temperature change with two seizures, 42 min apart, in one patient. Onset of seizure of right posterior quadrant onset indicated by the vertical dashed line. temperature probe in right ear. X-axis: time in minutes before and after seizure onset. Y-axis: epitympanic temperature in °C.

**Fig. 4:**
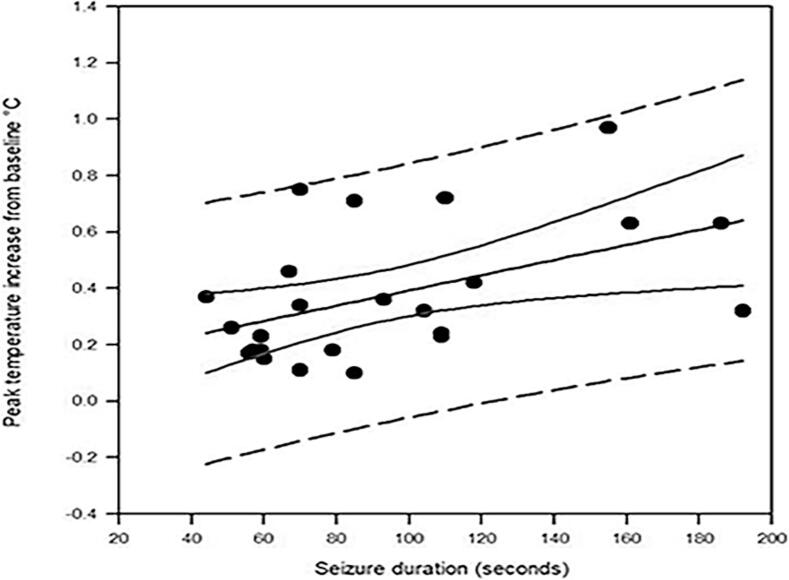
Linear regression analysis. Peak *peri*-ictal temperature change from baseline versus seizure duration. The linear regression line, confidence and prediction intervals (dashed lines) are depicted.

Most seizures had a ramp-like increase in temperature (Fig. 1a and 2). Fig. 1b shows a single channel of compressed EEG recorded concurrently with the temperature trace shown in Fig. 1a indicating that this seizure onset occurred around 12 min after a rise in temperature occurred. With some seizures the initial rise in temperature was followed by a plateau where the temperature rise remained relatively unchanged until seizure onset (Fig. 2, seizures E and F). The abrupt drops in temperature (Fig. 1a and in Fig. 2, traces A, C, G, H, and I) around seizure onset are related to the temperature probe being dislodged from the ear canal and temperature falling to ambient room temperature. The contours of the *peri*-ictal temperature change showed a close similarity in patients where more than one seizure was captured (Fig. 3). The magnitude of *peri*-ictal peak temperature change from baseline was associated with longer seizure duration (linear regression, p = 0.017) (Fig. 4).

In one patient, at seizure onset, three of four seizures of bilateral parietal onset did not have a pre-ictal rise in temperature, relative to baseline, of 0.1 °C or greater. However, with these seizures, the temperature peaked immediately after seizure onset with a temperature differential, relative to baseline, of greater than 0.1 °C. A fifth seizure had no discernable rise in temperature above baseline.

In one patient with multifocal onset seizures, two of three seizures of right posterior quadrant onset had a pre-ictal temperature rise and a third seizure, with onset over the frontal region, had no detectable pre-ictal temperature change.

## 4 Discussion

We have shown that there is a pre-ictal temperature increase, relative to the preceding interictal baseline temperature, and this increase can be detected non-invasively in semi-ambulatory patients with focal onset seizures. The epitympanic temperature changes reflect a pre-ictal rise in brain temperature. The magnitude of the epitympanic temperature change is comparable to brain temperature increases of approximately 0.3 °C recorded in rodents and primates during focal seizures (Torres et al., 2023, Yang et al., 2002). In patients with focal seizures, the pre-ictal duration of epitympanic temperature aligns with the previously reported duration of pre-ictal increases in cerebral blood flow and oxygenation that ranged from five to twelve minutes as seen in direct recordings over cerebral cortex, SPECT, fMRI and near-infrared spectroscopy studies (Adelson et al., 1999, Baumgartner et al., 1998, Seyal, 2013, Weinand et al., 1997).

In non-anesthetized, post-neurosurgery patients, brain temperature changes were shown to correlate significantly with changes in tympanic membrane temperature (Mariak et al., 1994). Tympanic membrane temperature is closely correlated with brain temperature (Dickey et al., 1970) as the middle ear is heated passively by the brain (Cherbuin and Brinkman, 2007). Tympanic membrane temperature is likely altered by changes in venous outflow temperature from the brain given the proximity of the middle ear to the jugular bulb (Mariak et al., 1994). Hippocampal temperatures increased by 0.1 to 0.5 °C after electrical stimulation of the perforant path (Moser and Mathiesen, 1996). As cerebral activity increases, cerebral blood flow also increases, resulting in a subsequent decrease in brain temperature (Sukstanskii and Yablonskiy, 2006) as carotid blood is cooler than cerebral tissue (Kiyatkin et al., 2002).

Since the thermistor used in our study was not sealed within the ear canal and was at some distance from the tympanic membrane, the recorded epitympanic temperatures in this study were lower than would be expected in direct measurements from the tympanic membrane and would be partially affected by the ambient room temperature.

The pathophysiological basis underlying the correlation between the magnitude of the peak *peri*-ictal temperature rise and seizure duration is undetermined. A similar correlation between temperature rise and seizure duration was reported in a rodent model of 4-aminopyridine-induced focal seizures and the possibility of focal seizures generating a sufficient rise in temperature to effect their own prolongation was hypothesized in that report (Yang et al., 2002).

There are technical challenges in continuously recording of ear canal temperatures over prolonged periods in the EMU. Patient movement and discomfort result in intermittent dislodging of the probe from the ear canal thus precluding complete assessment of circadian temperature variations. Given these limitations, we did not study other factors that may result in diurnal temperature fluctuations or temperature fluctuations related to non-seizure patient activities. Non-seizure related temperature changes could conceivably equal or exceed the pre-ictal temperature rise detected in the ear canal and will need to be studied with methodology permitting reliable recordings over prolonged periods. Any circadian temperature variations may have a longer time course than the duration of the pre-ictal temperature rise. In this group there was a relatively large range of baseline interictal temperatures across patients (Table 1). Several factors may have contributed the interictal temperature variability between patients such the distance of the probe from the tympanic membrane, ambient room temperature, and circadian temperature variations.

Two of the patients in this dataset had seizures of extratemporal onset. These patients did not have a robust and consistent pre-ictal temperature rise with temperatures peaking after seizure onset or had no detectable change in temperature. These data suggest that with some extratemporal seizures, pre-ictal epitympanic temperature recordings may be relatively insensitive to pre-ictal brain temperature changes.

Refinements in non-invasive methods for measuring pre-ictal brain temperature changes could lead to development of a tool for seizure prediction in ambulatory patients with refractory epilepsy. Better tolerated extracranial sensors for long-term use, with greater sensitivity to temperature change, may allow for long-term use in ambulatory patients. In patients undergoing intracranial EEG monitoring in the EMU, the addition of intracranially placed thermocouples for the duration of the VET (days to a couple of weeks) could provide robust and prolonged temperature information allowing for assessment of non-seizure related temperature variations. In ambulatory patients with permanently implanted intracranial neuromodulation devices such as responsive neurostimulation (RNS), the addition of intracranial temperature sensors may similarly provide a warning of an impending clinical seizure. Current RNS technology does not provide such information. A reliable pre-ictal warning will allow for responsive automated focal administration of a short-acting antiseizure drug or automated focal cooling of the seizure focus to stop an impending seizure.

## 5 Conclusion

In patients with focal onset seizures, there is a rise in pre-ictal temperature relative to the preceding interictal temperature. These temperature increases exceeding 0.3° C can be detected non-invasively in the epilepsy monitoring unit and may be present for greater than 1000 s prior to electrographic seizure onset. Our exploratory findings provide information that should lead to development of temperature-based technology for a non-invasive seizure warning device.

## Funding

Department of Neurology, University of California, Davis.

## Declaration of competing interest

The authors declare that they have no known competing financial interests or personal relationships that could have appeared to influence the work reported in this paper.

## Data Availability

The data that support the findings of this study are available from the corresponding author upon reasonable request.
